# Brain volume is a better biomarker of outcomes in ischemic stroke compared to brain atrophy

**DOI:** 10.3389/fstro.2024.1468772

**Published:** 2024-10-29

**Authors:** Kenda Alhadid, Robert W. Regenhardt, Natalia S. Rost, Markus D. Schirmer

**Affiliations:** Department of Neurology, Massachusetts General Hospital, Harvard Medical School, Boston, MA, United States

**Keywords:** arterial ischemic stroke, brain volume, brain parenchymal fraction, BPF, modified Rankin Scale, mRS

## Abstract

**Objective:**

This study aimed to determine whether brain volume at the time of ischemic stroke injury is a better biomarker for predicting functional outcomes than brain atrophy.

**Background:**

Brain parenchymal fraction (BPF) has been used as a surrogate measure of global brain atrophy and a neuroimaging biomarker of brain reserve in studies evaluating clinical outcomes after brain injury. Brain volume itself is affected by natural aging, cardiovascular risk factors, and biological sex, among other factors. Recent studies have shown that brain volume at the time of injury can influence functional outcomes, with larger brain volumes being associated with better outcomes.

**Methods:**

Acute ischemic stroke cases at a single center between 2003 and 2011, with neuroimaging obtained within 48 h of presentation were eligible for the study. Functional outcomes represented by the modified Rankin Scale (mRS) score at 90 days post-admission (mRS score ≤ 2 deemed a favorable outcome) were obtained through patient interviews or per chart review. Deep learning–enabled automated segmentation pipelines were used to calculate brain volume, intracranial volume, and BPF on the acute neuroimaging data. Patient outcomes were modeled through logistic regressions, and a model comparison was conducted using the Bayes information criterion (BIC).

**Results:**

A total of 467 patients with arterial ischemic stroke were included in the analysis, with a median age of 65.8 years and 65.3% of the participants being male. In both models, age and a larger stroke lesion volume were associated with worse functional outcomes. Higher BPF and a larger brain volume were associated with favorable functional outcomes; however, a comparison of both models suggested that the brain volume model (BIC = 501) better explains the data than the BPF model (BIC = 511).

**Conclusion:**

The extent of global brain atrophy (and its surrogate biomarker BPF) has been regarded as an important biomarker for predicting functional post-stroke outcomes and resilience to acute injury. In this study, we demonstrate that a higher global brain volume at the time of injury better explains favorable functional outcomes, which can be directly measured in a clinical setting.

## 1 Introduction

With aging populations in the United States and around the world, along with an increased incidence of stroke among younger patient populations, the prevalence of arterial ischemic stroke is on the rise (World Health Organization, 2006; Feigin et al., 2014; Zhang et al., 2023). Understanding the determinants of post-stroke outcomes that may lead to functional independence is of great clinical, societal, and economic importance. Determining the most relevant clinical and imaging biomarkers for predicting functional outcomes is essential for developing targeted preventative and therapeutic approaches. Phenotypic information, such as age and lesion volume (Nakayama et al., 1994; Löuvbld et al., 1997; Thijs et al., 2000), have been utilized to model post-stroke outcomes; however, current models are insufficient to adequately explain clinically observed variations in outcomes.

Neuroimaging studies have started to reveal important factors related to clinical outcomes, such as white matter hyperintensity volume (Debette and Markus, 2010; Schirmer et al., 2019a; Hong et al., 2021; Ball et al., 2023). Additionally, studies have demonstrated that brain volume, specifically cortical volume, is related to an individual's cognitive abilities and intelligence, even when corrected for age, sex, and other collinearities (McDaniel, 2005; Deary et al., 2010; Van Essen et al., 2013; Pietschnig et al., 2015; Genç et al., 2018; Weerasekera et al., 2023). Many of these studies rely on high-resolution, often 1 mm^3^ T1-weighted imaging sequences. However, for acute ischemic stroke patients, prolonged imaging acquisitions can lead to a delay in time to treatment and thus negatively impact patients' outcomes. New developments in neuroimage analysis pipelines have enabled the assessment of volumetric neuroimaging markers at admission from clinical magnetic resonance imaging (MRI) scans (Schirmer et al., 2019a, 2020; Hoopes et al., 2022; Billot et al., 2023; Laso et al., 2024; Hoffmann et al., 2024). This has led to the identification of brain volume in stroke patients at admission as an independent biomarker of functional post-stroke outcomes (Schirmer et al., 2020; Sagnier et al., 2017; Oliveira et al., 2023; Schirmer et al., 2024). Volumetric brain studies often normalize each patient's brain volume by their intracranial volume, also known as brain parenchymal fraction (BPF), which can serve as a surrogate measure of global brain atrophy in cross-sectional studies (Vågberg et al., 2017; Bu et al., 2021; Luijten et al., 2022; Yazici et al., 2024). However, no consensus exists on the utility of non-normalized and normalized brain volume measurements.

In this study, we compare the utility of non-normalized and normalized brain volume in outcome modeling by building on advances in deep learning–enabled segmentation algorithms to estimate brain volume and BPF in a cohort of 476 acute ischemic stroke patients based on their acute clinical neuroimaging data acquired in the emergency department. Using multivariable logistic regression models of functional outcome, measured using the 90-day modified Rankin Scale (mRS) score, we compare the models including either BPF as a surrogate measure of brain atrophy or a volumetric measure of brain volume. We demonstrate that an individual's brain volume at the time of acute injury, rather than a measure of brain atrophy, is a better marker for modeling functional outcomes.

## 2 Materials and methods

### 2.1 Standard protocol approvals, registration, and patient consent

The use of human patients in this study was approved by our site's institutional review board, and informed written consent was obtained from all participating patients or their surrogates at the time of enrollment following the Declaration of Helsinki.

### 2.2 Study design, setting, and patient population

Patients older than 18 years of age presenting to the emergency department at our hospital between 2003 and 2011 with signs and symptoms of acute ischemic stroke (AIS) were eligible for enrollment. In this analysis, we included subjects with (a) acute cerebral infarct lesions confirmed by diffusion-weighted imaging (DWI) scans obtained within 48 h of symptom onset and (b) T2 fluid-attenuated inversion recovery (T2-FLAIR) sequences available for volumetric analyses. All clinical variables, including demographics and medical history, were obtained on admission. Patients and/or their caregivers were interviewed in person or by telephone 3 months after the acute clinical stroke presentation to assess functional outcomes (mRS score). If the patient could not be contacted, an mRS score was determined from a review of clinical evaluations.

The standard AIS imaging protocol at time of presentation and obtained within 48 h of symptom onset in the emergency department on a 1.5T Signa scanner (GE Medical Systems) included DWI with single-shot echo-planar imaging (1–5 B0 volumes, 6–30 diffusion directions with *b* = 1,000 s/mm^2^, 1–3 averaged volumes) and T2-FLAIR imaging [Repetition time (TR) 5,000 ms, minimum Echo time (TE) of 62–116 ms, Inversion time (TI) 2,200 ms, Field-of-view (FOV) 220–240 mm]. DWI data sets were assessed and corrected for motion and eddy current distortions (Sorensen et al., 1999). Acute infarct volume was manually assessed on DWI (DWIv). A manual estimate of ICV was calculated on T1 sagittal sequences, where available [median (interquartile range, IQR) TR 400 (400–450) ms, TE 14 (10–14) ms, and FOV 240 (240–240) mm], using a previously validated method (Ferguson et al., 2005).

### 2.3 Automated brain and intracranial volume estimation

**Figure 1:**
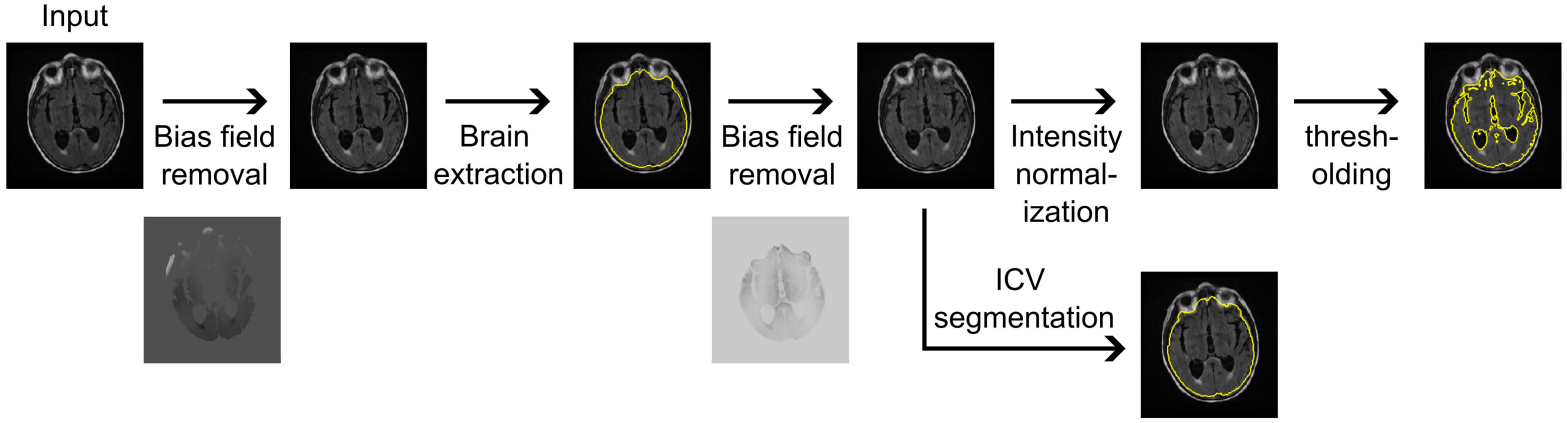
Magnetic Resonance imaging CLinical resOlution brain VolumEtRics (MR-CLOVER) pipeline for generating brain volume and intracranial volume (ICV) segmentations and volume estimations using clinical fluid-attenuated inversion recovery sequences obtained at hospital admission.

The brain volume and intracranial volume (ICV) estimations were calculated in a standardized, automated process utilizing the available FLAIR imaging data. Each patient image first underwent N4 bias field correction (Tustison et al., 2010), followed by brain extraction using synthstrip (Hoopes et al., 2022). The estimated brain mask was utilized in a secondary N4 bias field correction, after which the image underwent intensity normalization using a mean shift algorithm, and normal-appearing white matter was set to an intensity of 0.75. Subsequently, each image underwent thresholding at 0.375 to extract an estimate of total brain volume, given by combined gray and white matter volume, following our previously published approach (Schirmer et al., 2020). ICV masks were estimated based on the segmentation results from synthseg (Billot et al., 2023), utilizing the bias field corrected image as input. Figure 1 presents an overview of the MRI CLinical resOlution brain VolumEtRics (MR-CLOVER) pipeline. Both synthstrip and synthseg are publicly available as part of Freesurfer (Fischl, 2012), and the MR-CLOVER pipeline is also publicly available^1^.

### 2.4 Statistical analysis and model description

Before analysis, each mask underwent manual quality control by visual inspection. For each patient, brain volume and ICV were determined by multiplying the number of voxels by the corresponding voxel size. Automated and manual estimates of ICV were compared using a linear model without intercept, reporting the coefficient.

We calculated BPF for each patient as the ratio of brain volume to intracranial volume, which was subsequently logit-transformed. Age and brain volume were utilized in the model in units of decade and dm^3^, respectively, to avoid modeling issues due to scale. Patient outcome was encoded as functional independence (mRS score ≤ 2) and moderate to severe disability (mRS score >2). Patient outcome was then modeled through logistic regressions, given as


$$\begin{array}{r} mRS\left(\;>\;2\;\right)\;\sim \;Age\;+\;Sex\;+\;HTN\;+\;DM2\;+\;Non-Smoker\\ +\;DWIv\;+\;X, \end{array}$$

where *X* was either BPF or brain volume, resulting in two models for comparison. A model comparison was conducted using the Bayes information criterion (BIC).

After model fit, we tested the model assumptions, that is, linearity in the logit for continuous variables, the absence of multicollinearity given by a variance inflation factor lower than 2, and a lack of strongly influential outliers. All statistical analyses were conducted using the computing environment R (R Core Team, 2024). Significance was set at *p* < 0.05.

## 3 Results

The clinical characteristics of the study cohort are described in Table 1. The cohort had a median age (IQR) of 65.8 (55.3, 76.3) years, 65.3% were male, 69.7% had a diagnosis of hypertension, and 24.8% of patients had a bad outcome with an mRS score >2. All brain and ICV masks passed the quality control, and the volumes are summarized in Table 1. Manual and automated ICV estimates showed good agreement (coefficient ± standard error; β = 0.944 ± 0.002), enabling us to utilize the automated ICV estimates for further analyses.

**Table 1:** Characteristics of the cohort utilized in this study.

*N*	476
Age [years; median (IQR)]	65.8 (55.3, 76.3)
Sex (% male)	311 (65.3)
HTN (%)	332 (69.7)
DM2 (%)	96 (20.2)
Non-smoker (%)	286 (60.1)
Lesion volume [cc; median (IQR)]	2.2 (0.6, 12.7)
BPF [%; median (IQR)]	0.81 (0.77, 0.83)
Brain volume [cc; median (IQR)]	1,306.9 (1,190.9, 1,413.9)
mRS score >2 (%)	118 (24.8)

IQR, interquartile range; HTN, hypertension; DM2, diabetes mellitus type 2; BPF, brain parenchymal fraction.

**Figure 2:**
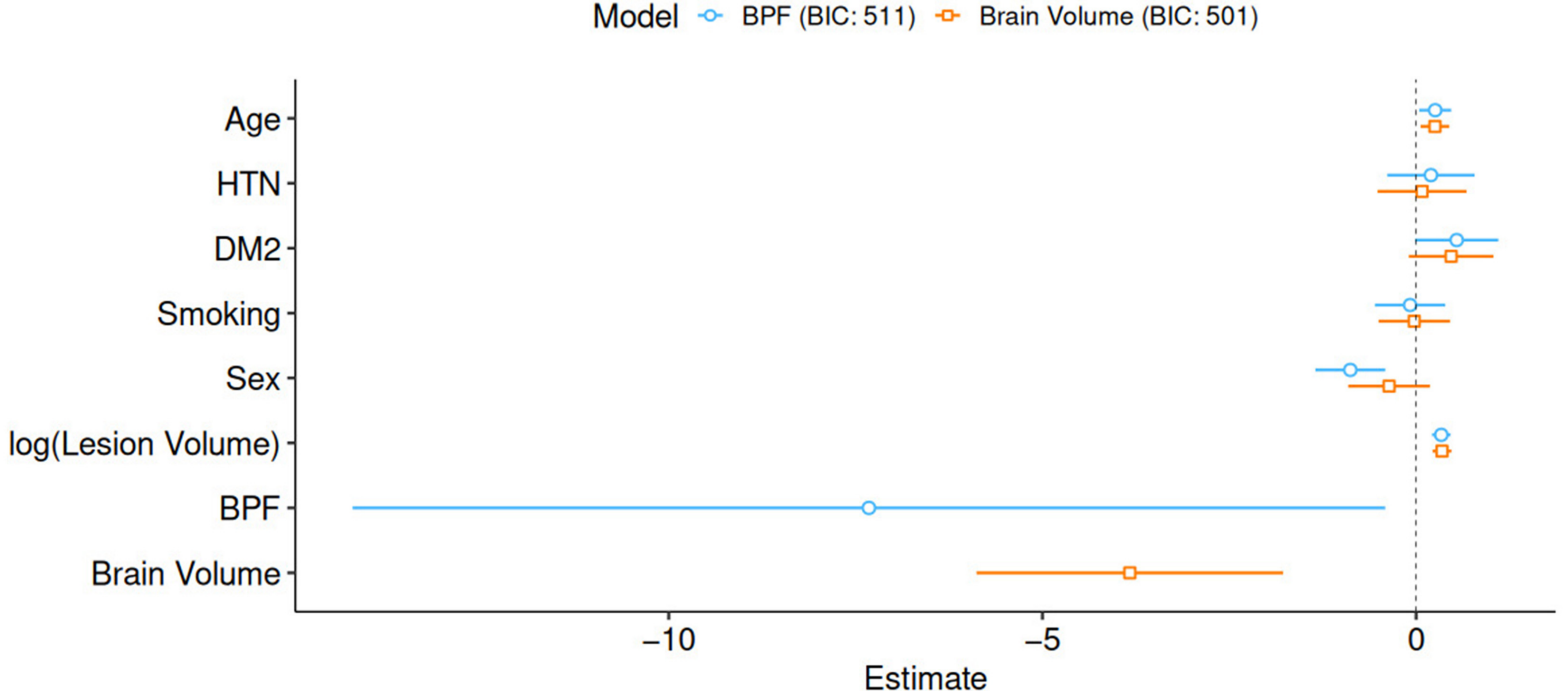
Graphical representation of parameter estimates including 95% confidence intervals. BIC, Bayes information criterion; HTN, hypertension; DM2, diabetes mellitus type 2; BPF, brain parenchymal fraction.

The parameters of both outcome models are summarized in Table 2 and Figure 2. All assumptions of the logistic regression models were fulfilled. In both models, older patients and patients with larger stroke lesion volume had worse outcomes. Male sex was only found to be significant in the BPF outcome model, where male patients demonstrated better functional outcomes. Both higher BPF, that is, less brain atrophy and higher brain volume were associated with better functional outcomes.

**Table 2:** Summary of model parameter estimates.

	**BPF**	***p***	**Brain volume**	***p***
Intercept	2.95	0.366	1.78	0.266
Age	**0.25**	**0.019**	**0.25**	**0.010**
Sex (M)	–**0.88**	**< 0.001**	−0.36	0.194
HTN	0.20	0.506	0.08	0.792
DM2	0.54	0.056	0.47	0.105
Non-smoker	−0.08	0.737	−0.02	0.919
log(lesion volume)	**0.34**	**< 0.001**	**0.35**	**< 0.01**
BPF	–**7.32**	**0.038**	–	
Brain volume	–	–	–**3.83**	**< 0.001**

HTN, hypertension; DM2, diabetes mellitus type 2; BPF, brain parenchymal fraction. Bold values are statistically significant.

Evaluating the BIC for both models resulted in values of 511 and 501 for the model based on BPF and brain volume, respectively. The comparison of both models suggests that the brain volume model explains the observed data better than the corresponding base model, with ΔBIC = 10.

## 4 Discussion

In this study, we highlight the significant role of brain volume and its association with functional outcomes after ischemic stroke. In a large cohort of AIS patients, we demonstrated that uncorrected brain volume at the time of injury is a better biomarker of stroke outcomes than brain atrophy. We derived brain volume and intracranial volume estimates automatically on clinical MRI sequences using a deep learning–enabled pipeline. This allowed this important parameter to be extracted from clinical imaging data obtained as the standard of care in the emergency department for patients with acute stroke presentations. Our results indicate that a larger brain volume at the time of the acute injury leads to better functional outcomes. In two models comparing brain volume and BPF, we determined a ΔBIC = 10, providing strong evidence that the brain volume model outperforms the BPF model (Raftery, 1995).

The relationship between larger brain volume and higher cognitive abilities has been consistently reported (McDaniel, 2005; Weerasekera et al., 2023; Royle et al., 2013), with more recent work delineating the underlying microstructural architecture observed in larger brain volumes that could explain this benefit. It is put forth that larger cortices benefit from the increased processing power of a higher number of neurons, with concomitant lower neurite density and orientation dispersion maximizing the network efficiency and reducing energy demand (Genç et al., 2018). Importantly, previous studies have shown that total brain volume is a significant determinant of functional (mRS) and patient-reported outcome after ischemic stroke (Schirmer et al., 2020; Oliveira et al., 2023). This may further relate to the concepts of brain and effective reserve, which aims to quantify the brain's ability to compensate for negative effects, such as sudden vascular events (Schirmer et al., 2024, 2019b; Stern et al., 2020). Our data show that brain volume without normalizing for intracranial volume was a better determinator of functional outcomes post-stroke.

In our model evaluating the relationship between BPF and functional outcomes at 90 days, male sex is significantly associated with a favorable functional outcome, and this is in line with previous studies showing that women are typically older at the time of stroke and endure worse functional outcomes post-stroke (Rexrode et al., 2022). However, sex becomes non-significant after including brain volume at the time of injury. This is likely explained by brain volume differences in the context of known anthropometric differences between men and women, which may account for the majority of sex-specific variation in the current data. Future large-cohort studies are needed to further disentangle sex-specific differences in patient outcomes.

Our study had limitations. Due to the AIS treatment timeline, a limited number of axial slices were obtained during MRI acquisition. These clinical scans lack isotropic resolution, experience partial volume effects, and prevent localized/regional brain volume estimates. However, the imaging data used reflects what is typically available during standard of care in acute stroke patients, in which rapidly estimating biomarkers is crucial for clinical decision-making. Additionally, examining the effects of lesion location, including the impact of strokes in eloquent vs. non-eloquent areas, was not performed. Lesion location and regional brain volume estimates have the potential to further refine outcome models in stroke and should be considered in future studies. Acknowledging that brain volume within 48 h of acute ischemic stroke might not fully represent stable conditions due to neuroinflammation and remodeling processes occurring in the acute phase after stroke is also important. This is compounded by potential confounding effects of acute edema, which can vary among patients and impact brain volume assessments. *Ad hoc* analysis showed a low correlation between brain volume and lesion volume (*r* = 0.1, *p* = 0.035), aligning with previous literature (Schirmer et al., 2020). In this study, no follow-up imaging data were available, but a detailed investigation into this aspect will be an important part of future research. Finally, acute treatments administered to stroke patients, particularly thrombolytic therapy in our cohort, are likely to influence outcomes. Imaging was conducted shortly after admission, mitigating the direct effects of treatment on brain volume. However, treatment, as well as other aspects such as risk factor control, neurorehabilitation, and other post-stroke interventions, will have a significant impact on outcomes; however, this information was not available. Future studies should consider these variables to refine outcome predictions.

Our study's strengths include the utilization of a large hospital-based cohort with clinical neuroimaging data available in the emergency department. Importantly, employing state-of-the-art clinical neuroimaging analysis methodologies enabled us to delve deeper into the associations with post-stroke outcomes at admission to the hospital, identifying neuroimaging biomarkers that can readily be assessed in the clinic. To the best of our knowledge, this study represents the first investigation into the benefit of utilizing non-normalized volumetric estimates of brain volume over measures of brain atrophy.

## 5 Conclusion

Our study provides strong evidence in a large cohort of stroke patients that brain volume at the time of injury is a better determinant of functional post-stroke outcomes than brain atrophy. Importantly, the presented analysis pipeline was based on clinical MRI data, offering the opportunity for immediate translation to a clinical tool that can quantify this biomarker at the initial point of care. This opens new avenues for expanding our knowledge on prognosticating outcomes in stroke populations.

## Data Availability

The data analyzed in this study is subject to the following licenses/restrictions. The authors agree to make the data, methods used in the analysis, and materials used to conduct the research available to any researcher for the express purpose of reproducing the results and with the explicit permission for data sharing by the local institutional review board. Requests to access these datasets should be directed to Markus D. Schirmer, mschirmer1@mgh.harvard.edu.

## Footnotes

^1^ https://github.com/mdschirmer/mr-clover
